# CU06-1004 as a promising strategy to improve anti-cancer drug efficacy by preventing vascular leaky syndrome

**DOI:** 10.3389/fphar.2023.1242970

**Published:** 2023-08-30

**Authors:** Songyi Park, Sunghye Lee, Dongyeop Kim, Hyejeong Kim, Young-Guen Kwon

**Affiliations:** ^1^ Department of Biochemistry, College of Life Science and Biotechnology, Yonsei University, Seoul, Republic of Korea; ^2^ Curacle Co., Ltd., Seoul, Republic of Korea

**Keywords:** IL-2 immunotherapy, permeability, vascular leaky syndrome (VLS), drug side effect, endothelial dysfunction blocker

## Abstract

**Background:** Interleukin-2 (IL-2) is the first cancer therapeutic agent with an immunomodulatory function. Although it has been experimentally proven to be effective against metastatic renal cell carcinoma and metastatic melanoma, the clinical application of high-dose IL-2 (HDIL-2) has been limited because of its short half-life and severe side effects, such as vascular leakage syndrome (VLS) or capillary leaky syndrome (CLS). However, methods for overcoming this issue have not yet been identified.

**Methods:** We discovered CU06-1004, an endothelial dysfunction blocker, through a previous study, and co-treated with IL-2 immunotherapy to confirm its inhibitory effect on HDIL-2-induced endothelial permeability. CU06-1004 was co-administered with HDIL-2 for 4 days in an *in vivo* mouse model. After drug injection, the mice were sacrificed, and Evans blue staining was performed.

**Results:** *In vitro*, HDIL-2 treatment decreased HUVEC stability, which was rescued by co-treatment with CU06-1004. In our mouse model, co-administration of CU06-1004 and HDIL-2 prevented HDIL-2-induced vascular leakage by normalizing endothelial cells. Notably, the HDIL-2 and CU06-1004 combination therapy considerably reduced tumor growth in the B16F10 melanoma mouse model.

**Conclusion:** Our data suggest that CU06-1004 acts as a potential anticancer drug candidate, not only by preventing HDIL-2-induced VLS but also by enhancing the anticancer effects of HDIL-2 immunotherapy.

## 1 Introduction

Cancer immunotherapy has indeed revolutionized anti-cancer drugs, and IL-2 is a key immunotherapeutic agent used to stimulate the immune system to attack cancer cells (Farkona et al., 2016; Zhao et al., 2019; Zhang and Zhang, 2020). It exhibits pleiotropic effects on the immune system, particularly its ability to promote the development of white blood cells and release chemicals that attract cancer-killing immune cells; these effects make it a valuable tool in the fight against cancer (Galli et al., 2020; Pena-Romero and Orenes-Pinero, 2022). High-dose IL-2 (HDIL-2) was approved by the FDA for the treatment of metastatic renal cell carcinoma in 1992 and of metastatic melanoma in 1998 (Payne et al., 2014; Alva et al., 2016).

However, its use was limited due to its short half-life and serious side effects (Skrombolas and Frelinger, 2014; Sun et al., 2019; Merchant et al., 2022). Similar to the commonly known side effects of anticancer drugs, IL-2 therapy causes a flu-like syndrome, fever, nausea, vomiting, and asthenia (Altun and Sonkaya, 2018; Mortara et al., 2018). However, the major side effect of HDIL-2, used for cancer regression, is vascular leaky syndrome (VLS) and capillary leaky syndrome (CLS) (Baluna and Vitetta, 1997; Sivakumar et al., 2013; Kim et al., 2014). Severe VLS is caused by HDIL-2 treatment, which increases vascular permeability and decreases microcirculatory perfusion (Moreno et al., 2006; Guan et al., 2012; Sivakumar et al., 2013). Ultimately, it causes extensive fluid retention in multiple organs, such as the lungs, liver, and heart, and can lead to pulmonary edema, liver cell damage, and cardiovascular failure (Krieg et al., 2010; Chen et al., 2018).

To overcome this effect, we administered combination therapy with CU06-1004, a previously known endothelial dysfunction blocker (Park et al., 2020; Bae and Kwon, 2022; Zhang et al., 2022). CU06-1004 sustains vascular stabilization and strengthens the endothelial barrier (Park et al., 2020). In addition, CU06-1004 inhibits vascular leakage by forming cortical actin rings via cAMP/Rac/cortactin (Kim D. Y. et al., 2020). It leads to the regulation of various factors such as vascular endothelial growth factor (VEGF), histamine, and thrombin (Kim Y. S. et al., 2020; Park et al., 2020). In a previous study, CU06-1004 reduced IL-1β-induced endothelial permeability and NF-κB activation, neurological deficits, cerebral infarction, and glial activation in an ischemic stroke mouse model (Kim D. Y. et al., 2020). The therapeutic effects of CU06-1004 have been demonstrated in various disease models, such as cancer, stroke, and diabetic retinopathy. Our cancer study showed that CU06-1004 induced tumor vessel normalization by enhancing junction proteins, pericytes, and smooth muscle actin and overcame tumor progression and treatment resistance (Park et al., 2020).

Notably, co-administration of CU06-1004 and IL-2 has shown potential in overcoming severe vascular leaky syndrome induced by HDIL-2 therapy. We overcame the decreased viability and increased permeability of endothelial cells induced by IL-2 in an *in vitro* HUVEC model by co-injection with CU06-1004. In addition, we established IL-2-induced side effects in an *in vivo* model and confirmed the reduction of side effects through combined administration. Additionally, HDIL-2 produced tumor suppression and reduced side effects in the B16F10 tumor-bearing mouse model. This suggests that CU06-1004 could serve as a potential anticancer drug candidate, not only by preventing HDIL-2-induced VLS but also by enhancing the anticancer effects of HDIL-2 immunotherapy.

## 2 Materials and methods

### 2.1 Cell lines and culture

Human umbilical vein endothelial cells (HUVECs) were purchased from Lonza (Basel, Switzerland). HUVECs were cultured in plates coated with 2% gelatin (Sigma-Aldrich) and endothelial cell basal medium-2 (Lonza) supplemented with EGM SingleQuots (Lonza) at 37°C in 5% CO_2._ B16F10 murine melanoma cells (kindly gifted by Prof. Sang-Jun Ha; Yonsei University, Seoul, Korea) were cultured in complete Dulbecco’s modified Eagle’s medium (DMEM; Hyclone; SH30022.01) supplemented with 10% fetal bovine serum (FBS; GE Healthcare UK Ltd.) and 1% penicillin/streptomycin (Gibco Laboratories) at 37°C in 5% CO_2_ incubator in a humidified atmosphere.

### 2.2 Mice

Male C57BL/6 mice, aged 6–7 weeks, were purchased from DBL Korea under semi-SPF conditions. All experiments were approved by the committee of Yonsei University (IACUC-A-202104-1252-01).

### 2.3 Drugs

Recombinant IL-2 immunotherapy (Recombinant human IL-2; 200-20) was purchased from Peprotech, Korea. CU06-1004 has been previously reported (Maharjan et al., 2013; Lee et al., 2014). To synthesize CU06-1004, a tetrahydropyran analog was prepared by reacting dihydropyran and pregnenolone in *p*-toluenesulfonic acid. After Wittig olefination with 4-(carboxybutyl) triphenylphosphonium bromide, the acid moiety was methylated using trimethylsilyl diazomethane. CU06-1004 was synthesized via tetrahydropyran deprotection and subsequent glycosidation with 4, 6-di-*O*-acetyl-2, 3-didieoxyhex-2-enopyran in the presence of an acid.

### 2.4 IL-2 and CU06-1004 treatment

Recombinant IL-2 drug was intraperitoneally (i.p.) injected with 75,000 U of three times a day for 3 consecutive days. On day 4, the mice received one injection. After 2 h, the mice were sacrificed. CU06-1004 was dissolved in 100 μL of olive oil (Sigma-Aldrich, St. Louis, MO), and a dose of 10 mg/kg was administrated by using oral gavage daily for the same duration as the IL-2 drug.

### 2.5 *In Vitro* cell cytotoxicity

Cell viability and proliferation were compared by 3-(4,5-dimethylthiazol-2-yl) -2,5-diphenyl tetrazolium-bromide (MTT) assay. HUVEC were seeded in 24-well plates (2 Χ 10^4^ cells/well). After treatment with CU06-1004 and IL-2, cells were maintained for 24 h in media containing 0.2% FBS. MTT (0.5 mg/mL) was added to each well, and cells were incubated at 37°C for 3 h. The supernatant was removed, and 200 μL DMSO + isopropyl alcohol was added to dissolve the formazan product. Absorbance, which is proportional to the number of living cells and proliferation rate, was measured at 540 nm on a microplate reader (FLUOstar Omega, BMG LABTECH). Data represent four independent experiments.

### 2.6 Immunofluorescence staining of human umbilical vein endothelial cells (HUVECs)

To examine vascular permeability, HUVEC were fixed in 4% paraformaldehyde for 10 min and permeabilized with 0.1% Triton X-100 in PBS for 15 min at room temperature. The cells were incubated with primary antibodies against VE-cadherin (1:200; Santa Cruz Biotechnology) at 4°C for 16 h. Cells were then incubated with secondary antibodies conjugated to Alexa Fluor 594 for 1 h at room temperature. Actin filaments were incubated with rhodamine-phalloidin (1:250; Molecular Probes) for 30 min. For nuclear staining, the cells were treated with DAPI (1:1000) for 20 min before mounting. Immunofluorescent images were obtained using a confocal microscope (Carl Zeiss 700, Germany).

### 2.7 Endothelial cell permeability assay

Human umbilical vein endothelial cells were seeded at a density of 4 × 10^5 cells/well onto 12-well Transwell semipermeable supports (0.4 μm pore size; Corning) coated with 1% gelatin. HUVEC were cultured in EC basal medium (EBM-2, CC-3156) containing EGM-2-kit (CC-4176) (Lonza Walkersville, Inc., MA, United States) and 10% FBS at 37°C in a 5% CO_2_ incubator in a humidified atmosphere. Upon confluence, the cells were starved in serum-depleted medium for 2 h and treated with 100 kilounits/mL IL-2 for 4 h. Endothelial cell permeability was confirmed using fluorescein isothiocyanate (FITC)-dextran fluorescein. FITC-dextran (30 mg/mL; Sigma-Aldrich) was added to the upper chamber and incubated for 30 min. The absorbance was measured at 492 nm (excitation) and 520 nm (emission) using a FLUOstar Omega microplate reader. The transendothelial electrical resistance (TEER) assay was performed using a chopstick electrode (World Precision Instruments STX2) with Millicell ERS-2 volt/Ω m (Millipore, MA, United States) and the results expressed as Ω × cm^2^.

### 2.8 *In vivo* tumor models

Tumors were subcutaneously implanted into the right flanks of 6- to 7-week-old C57BL/6 mice. Tumor volumes were measured every day according to formula (0.523 x (length x width^2^)). The drug was injected approximately 1 week after the tumor was implanted.

### 2.9 Evans blue staining

To analyze vascular permeability, mice were injected intravenously (i.v.) injected with 1% Evans blue dye (Sigma-Aldrich) diluted in 100 μL saline. Fifteen minutes later, mice were anesthetized with 2.5% avertin (Sigma-Aldrich) via intraperitoneal (i.p.) injection. And mice were perfused with 20 mL PBS, and tissues (Lung, Liver, Hand, Foot) were harvested, and dye was extracted in Formamide (500 μL, Junsei, Tokyo, Japan) overnight at 60°C. Dye concentrations were quantified by measuring absorbance at 620 nm. The content of Evans blue dye was determined by generating a standard curve from dye dilutions.

### 2.10 Immunofluorescence staining of tumor tissue

Mice were anesthetized with i. p. 2.5% avertin and then perfused with 50 mL PBS or saline via the left ventricle of the heart. Whole tumors were collected, fixed with 4% paraformaldehyde (PFA) for 16 h (h), dehydrated in a 15% sucrose solution, and followed by a 30% sucrose solution until tumors sank to the bottom of the container. Mouse tumor tissues were sectioned 20–30 μm thick using a cryostat (Leica, Wetzlar, Germany). One of every 7 to 10 slices was collected. Sections were stored at −80°C. To examine increased T cells in the tumor, CD8 (Abcam; ab22378; 1:200) was performed at 4°C for 16 h. After washing, slides were incubated with the appropriate Alexa-Flour 488-conjugated secondary antibodies (1:500) at RT for 1 h. Immunofluorescence was imaged using confocal microscopy (Carl Zeiss 700, Germany). Quantification of fluorescence intensity and cell counting was performed using Image J (NIH) or Photoshop version CS6 (Adobe Systems, San Jose, CA).

### 2.11 Enzyme-linked immunosorbent assay

To analyze cytokine levels between drug-treated groups, protein was collected from each Mouse serum. Quantification was performed with BCA protein reagent (SMART™ BCA Protein Assay Kit Solution A and B; iNtRON BIOTECHNOLOGY; 21071) and RIPA buffer assay (cOmplete ULTRA Tablets; Roche). IFNγ (Mouse IFN-gamma Quantikine ELISA; R&D Systems; MIF00) was measured by ELISA kit.

### 2.12 Statistical analysis

Data are presented as mean ± standard error of the mean (SEM). All statistical analyses were performed using GraphPad Prism (version 8; GraphPad Software, La Jolla, CA). The mean difference between groups was also analyzed by one-way ANOVA. *p < 0.05; **p < 0.01; ***p < 0.001; ****p < 0.0001. ns, not significant.

## 3 Results

### 3.1 CU06-1004 alleviates vascular hyper-permeability by preventing the reduction of endothelial cell viability by IL-2

**FIGURE 1:**
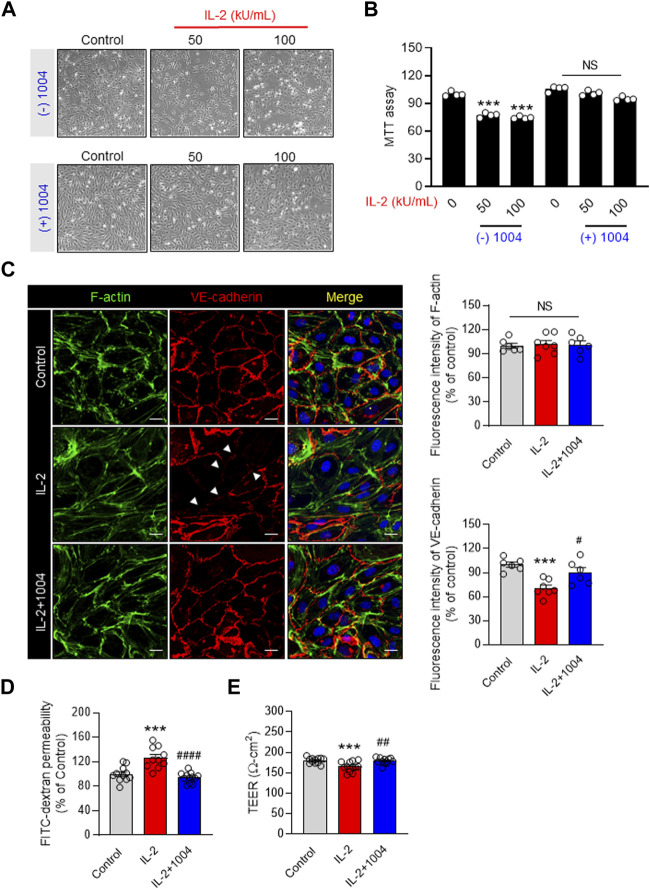
EC dysfunction blocker CU06-1004 improves the decrease in IL-2-induced endothelial cell viability and permeability. **(A, B)** HUVEC MTT assay was performed using a 24-well cell culture plate. After treatment with CU06-1004 (10 mpk) and IL-2 (100 kU/mL), the cells were incubated in a medium containing 0.2% FBS for 24 h. Data represent four independent experiments. Statistical analysis by one-way ANOVA with Tukey’s multiple comparisons. **(C)** IL-2 and CU06-1004 were administered after HUVEC starvation. Subsequently, the cells were fixed, permeabilized, and stained for adherent junction marker and F-actin. Green, F-actin; Red, VE-cadherin; blue, DAPI staining. *n* ≥ 3 independent experiments. **(D)** HUVEC were starved and treated with CU06-1004 and IL-2 for 4 h. Next, FITC-dextran (30 mg/mL; Sigma) was added to the upper chamber and incubated for 30 min. Absorbance was measured at 492 nm (excitation) and 520 nm (emission) using a FLUOstar Omega microplate reader. *n* ≥ 3 independent experiments. **(E)** The TEER assay was performed using a chopstick electrode (World Precision Instruments STX2) with Millicell ERS-2 volt/Ω m (Millipore, MA, United States) and given in ohm cm squared. *n* ≥ 3 independent experiments. Statistical analysis by one-way ANOVA with Tukey’s multiple comparisons. *p < 0.05; **p < 0.01; ***p < 0.001. ns, not significant. Data are presented as ± SEM.

IL-2 immunotherapy causes endothelial dysfunction, eventually inducing VLS. Therefore, we co-injected IL-2 and CU06-1004 to resolve the IL-2-induced side effects in endothelial cells. In previous studies, CU06-1004 has been reported as a blocker of endothelial dysfunction (Kim D. Y. et al., 2020; Kim Y. S. et al., 2020; Park et al., 2020; Bae et al., 2021). We first tested cell viability in the IL-2-alone group and the IL-2- and CU06-1004- combination group in HUVEC. As a result of the MTT assay to compare cell viability, the number of HUVECs was decreased in the IL-2 alone group. However, it was confirmed that the number of HUVECs in the IL-2 and CU06-1004 combination group was increased compared to IL-2 alone (Figures 1A, B). Therefore, we analyzed whether IL-2-induced permeability could be reduced by increasing cell viability by CU06-1004. It has been reported that stimulation with IL-2 disrupts interactions between adhesive junction (AJ) proteins, alters cell morphology and creates gaps between adjacent cells. Therefore, we performed VE-cadherin and F-actin immunostaining in the IL-2 alone and IL-2 and CU06-1004 combination group. At the cell borders, AJ proteins from normal HUVEC formed a linear pattern. Co-administration of CU06-1004 restored the linear pattern that was collapsed by IL-2 alone. Furthermore, in the IL-2 alone group, it was seen that the stress fiber was relatively increased compared to the normal group. However, the combination group with CU06-1004 showed no increase in stress fibers compared to the IL-2 alone group (Figure 1C). Additionally, We measured TEER and FITC-dextran in the HUVEC monolayers to determine endothelial barrier integrity and permeability. TEER decreased, and CU06-1004 changed the IL-2-induced hyper-permeability (Figures 1D, E). Ultimately, Our results showed that CU06-1004 suppressed the IL-2-induced negative effects by protecting the viability and reducing the permeability of endothelial cells.

### 3.2 Treatment of CU06-1004 ameliorates vascular leakage by HDIL-2 immunotherapy *in vivo*

**FIGURE 2:**
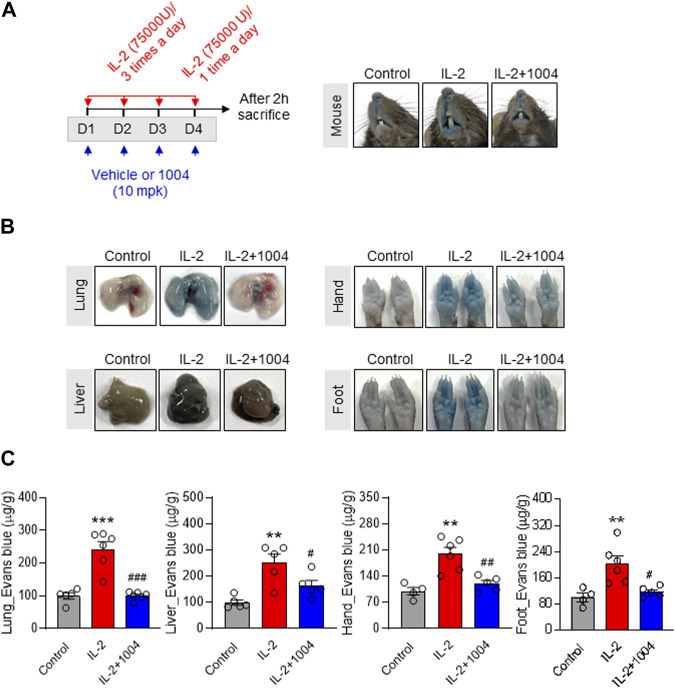
CU06-1004 alleviates IL-2-induced vascular leaky syndrome through Evans blue staining in normal mouse model. **(A)** Schematic depicting the schedule of IL-2 and CU06-1004 treatments in normal mice. Treatments were performed under the condition of IL-2-induced vascular leaky syndrome. Groups of four to five mice were injected i. p. with 75,000 U of IL-2 or PBS as a control three times a day for 3 consecutive days. On day 4, the mice received one injection, and 2 h later, they were injected. They were orally injected with 10 mpk (10 mg/kg) CU06-1004 daily. After 2 h, the mice were sacrificed. **(B–C)** Vascular permeability was quantified by i. v. administration of Evans blue dye. After dye administration, the mice were perfused with PBS, tissues were harvested, and dye extracted in formamide overnight. Dye concentrations were quantified by measuring absorbance at 620 nm. The content of Evans blue dye was determined by generating a standard curve from dye dilutions. **(**B) The representative image shows Evans blue staining in a normal mouse model after drug treatment. **(C)** The representative graph shows the percentages of VLS level by Evans blue staining. *n* = 4-6 per group. Statistical analysis by one-way ANOVA with Tukey’s multiple comparisons. *p < 0.05; **p < 0.01; ***p < 0.001. ns, not significant. Data represent ± SEM.

We previously showed that CU06-1004 protects against IL-2-induced endothelial damage. To confirm the reduction in vascular leakage in tissues by CU06-1004, we conducted Evans blue staining in normal mice by injecting the dye into the tail vein and measuring the amount of dye outflowing from the vasculature. IL-2 stimulation significantly induced vascular leakage in the tissues and skin, and IL-2-induced hyperpermeability was reduced owing to leakage blocking by CU06-1004 (Figures 2A–C). This result suggests the possibility of improving the VLS or CLS induced by HDIL-2 through combination therapy with CU06-1004.

### 3.3 CU06-1004 improves IL-2-induced VLS in the B16F10-bearing mouse model

**FIGURE 3:**
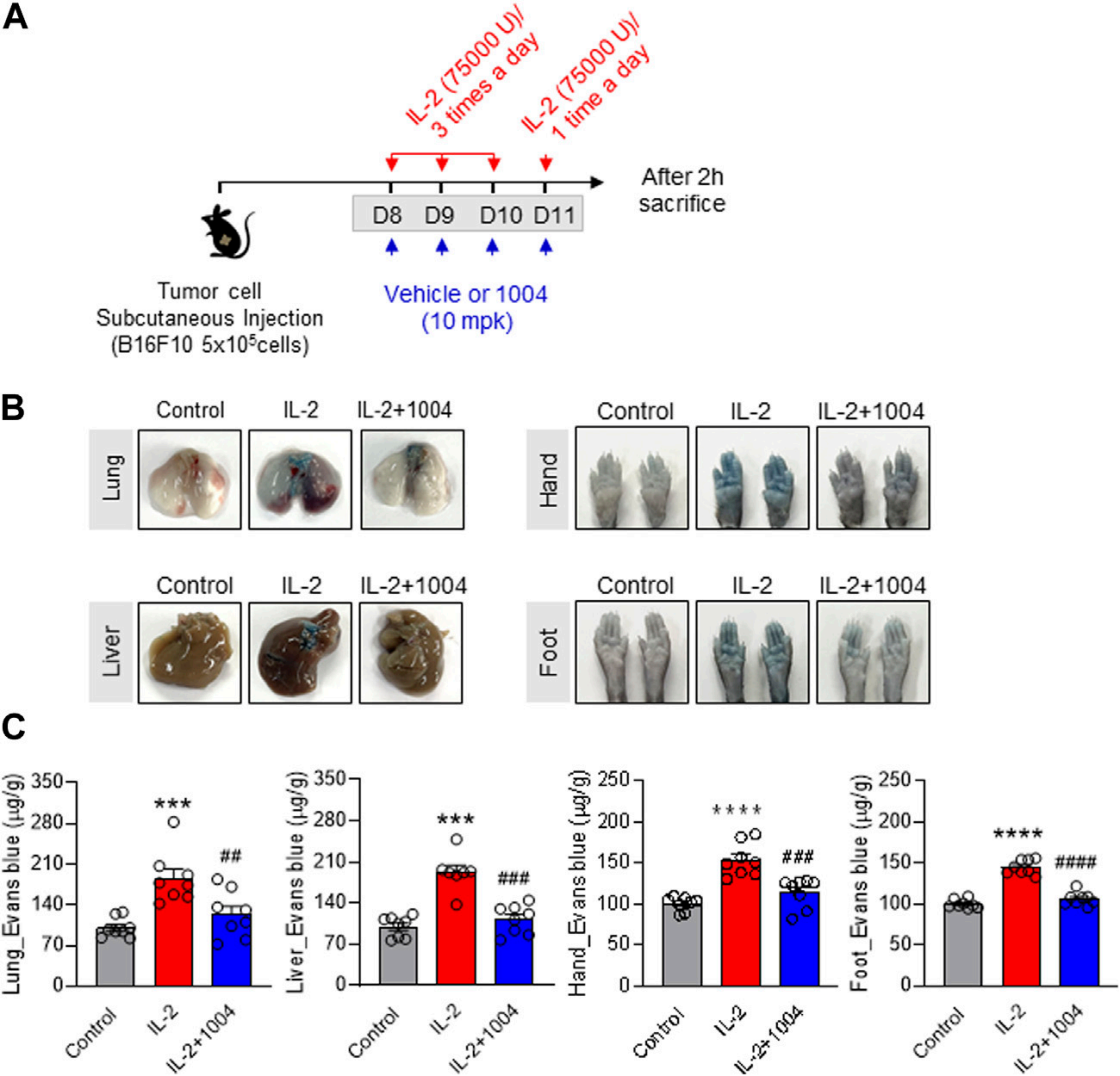
CU06-1004 alleviates IL-2-induced vascular leaky syndrome in B16F10 tumor-bearing model. **(A)** Schematic depicting the schedule of combination therapy in a B16F10 tumor-bearing mouse. **(B–C)** Vascular permeability was quantified by i. v. administration of Evans blue dye. After dye administration, mice were perfused with PBS, tissues were harvested, and dye extracted in formamide overnight. Dye concentrations were quantified by measuring absorbance at 620 nm. The content of Evans blue dye was determined by generating a standard curve from dye dilutions. **(B)** The representative image shows Evans blue staining in B16F10 tumor-bearing model after drug treatment. **(C)** The representative graph shows the percentages of VLS level by Evans blue staining. *n* = 8 per group. Statistical analysis by one-way ANOVA with Tukey’s multiple comparisons. *p < 0.05; **p < 0.01; ***p < 0.001. ns, not significant. Data represent ± SEM.

In a previous result, IL-2-induced vascular leakage in normal mice was observed through Evans blue staining, and it was found that co-administration with CU06-1004 improved this effect. Therefore, in Figure 3 of our study, we aimed to observe the degree of vascular leakage by IL-2 and CU06-1004 in the B16F10-bearing mouse model using Evans blue staining. To compare the degree of vascular leakage between groups, the amount of Evans blue dye was measured in Lung, Liver, Hand, and Foot. The results showed that vascular leakage in the IL-2 alone group was significantly increased compared to the control group, while it was decreased in the combination group with CU06-1004 compared to the IL-2 alone group (Figures 3A–C). These results demonstrated that the inhibition of vascular leakage in the tumor-bearing mouse model was the result of CU06-1004 injection, and emphasized that the side effects of IL-2 drugs could be improved by co-administration with CU06-1004.

### 3.4 Combination therapy maintains the tumor-killing effect in the B16F10-bearing mouse model

**FIGURE 4:**
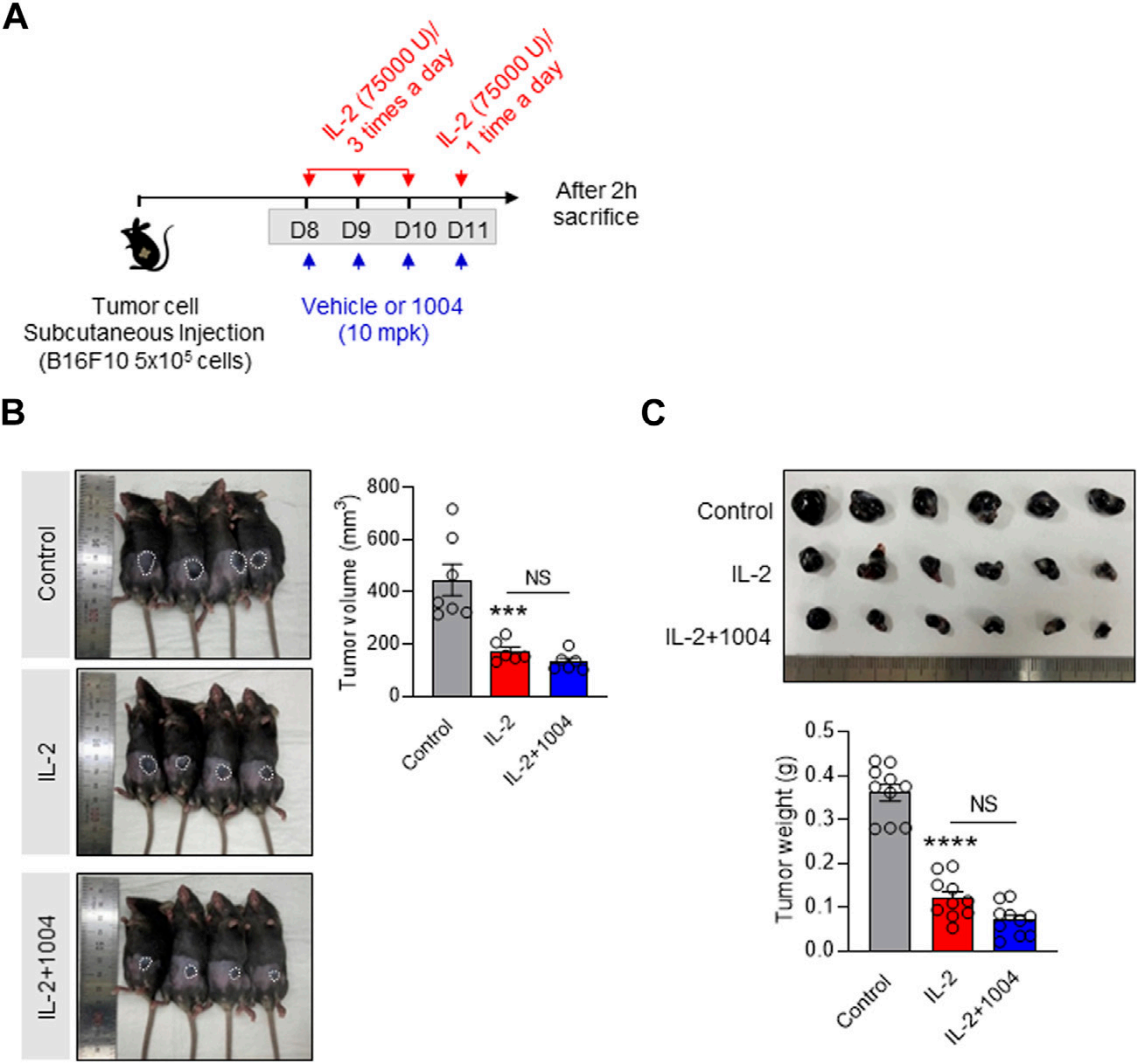
Combination of CU06-1004 and IL-2 decreased B16F10 melanoma growth and sustained IL-2 immunotherapy efficacy. **(A)** Schematic depicting the schedule of combination therapy in a B16F10 tumor-bearing mouse. B16F10 tumor cells (5 × 10^5^ cells/mouse) were injected subcutaneously into the right flank of C57BL/6 mice. The tumor-bearing mice were treated with IL-2 and CU06-1004 after tumor inoculation (tumor size <100 mm^3^). Groups of four to five mice were injected i. p. with 75,000 U of IL-2 or PBS as a control three times a day for 3 consecutive days. On day 4, the mice received one injection, and 2 h later, they were sacrificed. **(B)** Tumor volume (*n* = 6-7 per group) and **(C)** weight (*n* = 10) were measured from each group of mice. Statistical analysis by one-way ANOVA with Tukey’s multiple comparisons. *p < 0.05; **p < 0.01; ***p < 0.001. ns, not significant. Data are presented as ± SEM.

Our results to date have demonstrated that CU06-1004 can reduce IL-2-induced vascular leakage in a tumor-bearing mouse model. However, to consider its potential application in cancer patients, it is crucial to prove its tumor suppression effect in combination therapy. To show this, we monitored the size and weight of tumors in three groups: the control group, the IL-2 alone group, and the combination group with CU06-1004 (Figure 4A). Upon analysis, we found that the tumor size and weight in the IL-2 alone group were significantly reduced compared to the control group, indicating the efficacy of IL-2 in tumor suppression. Interestingly, in the combination group with CU06-1004, we also observed a significant reduction in tumor size and weight compared to the control group (Figures 4B,C). This intriguing finding suggests that the co-administration of CU06-1004 with IL-2 not only maintains the tumor suppression effect of IL-2 but also potentially enhances it. The data obtained from this study further supports the therapeutic potential of the combination therapy, indicating that CU06-1004 may complement the anti-cancer efficacy of IL-2 treatment.

### 3.5 CD8^+^ T cells are increased in CU06-1004- and IL-2-injected group

**FIGURE 5:**
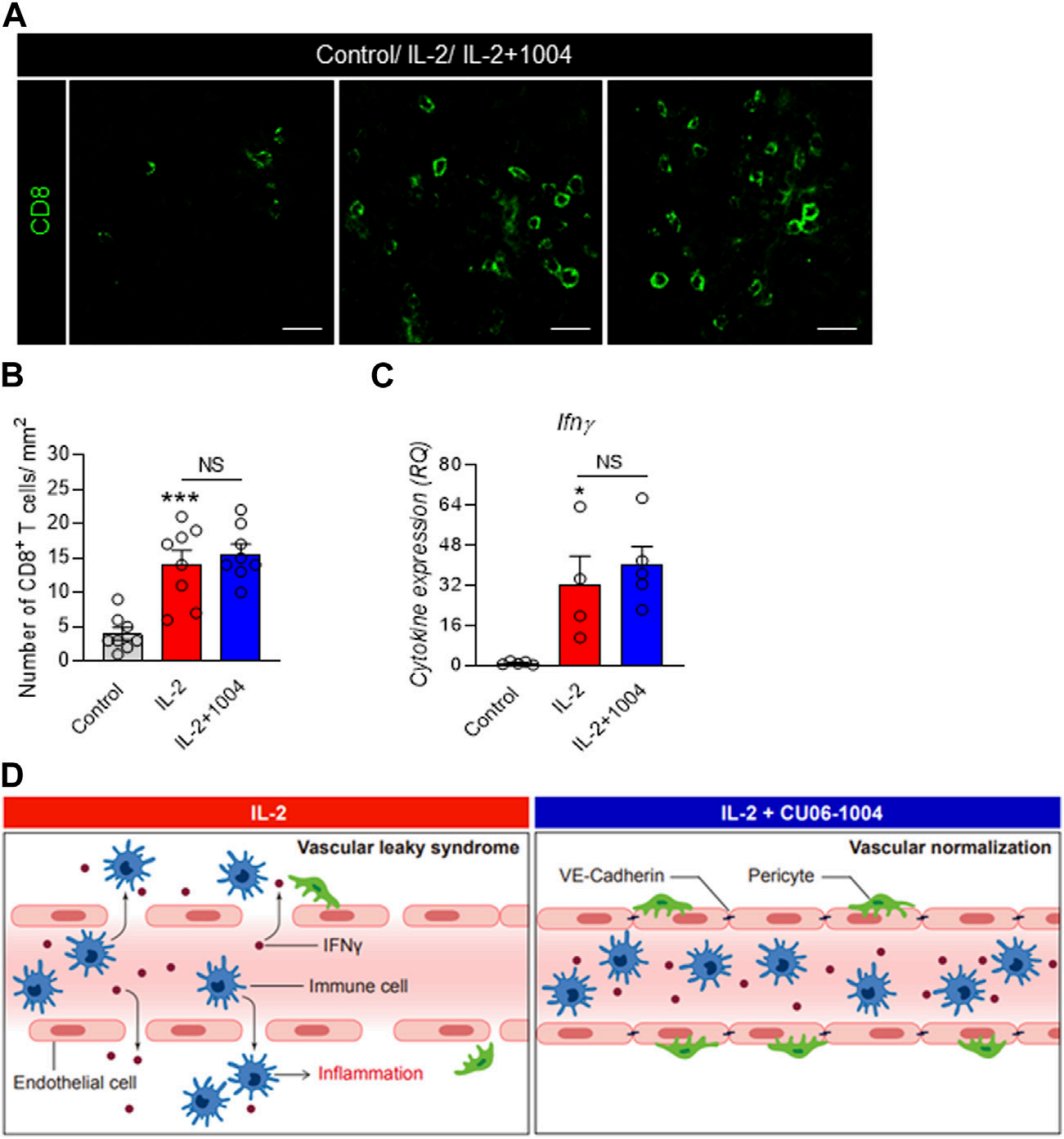
IL-2 and CU06-1004 combination therapy changes T cell infiltration and pro-inflammatory cytokine level. **(A)** Immunofluorescence staining data showed the accumulation of CD8^+^ T cells in the tumor site in the combination treatment group. Immunofluorescence was imaged via confocal microscopy (Carl Zeiss 880, Germany). **(B)** Representative graph showing the percentages of CD8^+^ T cells. *n* = 8 per group. Statistical analysis by one-way ANOVA with Tukey’s multiple comparisons. **(C)** ELISA demonstrated a significant difference in the serum levels of IFNγ. n = 4-5 per group. Statistical analysis by one-way ANOVA with Tukey’s multiple comparisons. *p < 0.05; **p < 0.01; ***p < 0.001. ns, not significant. Data are presented as ± SEM. **(D)** In the absence of CU06-1004, IL-2 induced endothelial cell damage and vascular permeability. However, injection of a combination of IL-2 and CU06-1004 maintained the endothelial cell viability and decreased the vascular permeability.

Next, in order to identify the number of immune and inflammatory cells in the tumor microenvironment, we conducted immunofluorescence staining of CD8^+^ T cells with cytotoxic abilities. Interestingly, when the tumor size was reduced by the IL-2 drug, the number of immune cells infiltrated into the tumor was significantly increased. However, there was no significant difference between the IL-2 alone group and the CU06-1004 co-administration group (Figure 5A). Additionally, to compare the cytokine changes caused by the increased CD8^+^ T cells, we confirmed the expression of IFNγ in mouse serum between the groups by ELISA. As a result, the expression of IFNγ was significantly increased in both IL-2 injected groups. These results were proportional to the increase in the number of CD8^+^ T cells with cytotoxic capability in tumors (Figure 5B). In summary, tumor size reduction by IL-2 drugs is expected to be related to the number of CD8^+^ T cells in the tumor and the expression of pro-inflammatory cytokines.

## 4 Discussion

Recently, human cancer treatment has developed rapidly with the targeting of immune response ‘checkpoints’ using cytotoxic T lymphocytes (Zhang et al., 2018; Waldman et al., 2020; Raskov et al., 2021). Regulation by T cells has a surprising effect on tumor regression by releasing cellular immune response, enabling long-term treatment (Walsh et al., 2019; Waldman et al., 2020). Nevertheless, according to a recent report, immunotherapies such as IL-2 cause fatal and serious side effects related to endothelial disorders (Li et al., 2017; Mortara et al., 2018). High doses of IL-2 therapeutics are correlated with treatment success, but lower doses of IL-2 reduce side effects and responses (Davar et al., 2017). High doses of IL-2 therapeutics are correlated with treatment success, but lower doses of IL-2 reduce side effects and responses (Rosenberg et al., 1994). Therefore, we co-administered CU06-1004, an endothelial dysfunction blocker, to suppress VLS (CLS) and cytokine ‘storm’, the most serious side effect of HDIL-2 therapy (Fig.6) (Skrombolas and Frelinger, 2014).

First, we performed an MTT assay in HUVEC to confirm that CU06-1004 recovered IL-2-induced toxicity at the cellular level. Depending on the concentration of IL-2, endothelial cells were separated from the extracellular matrix and intercellular junctions were degraded, leading to pores. However, the co-administration of CU06-1004, which induces endothelial cell stabilization and normalization, inhibited IL-2-induced damage and apoptosis. In addition, previous studies have reported that IL-2 increases endothelial cell permeability, which induces hypoalbuminemia in patients (Xie et al., 2012; Zloza et al., 2014; Soeters et al., 2019). This hypothesis is consistent with the results of our previous permeability assays. To achieve endothelial cell integrity, the cytoskeletal tissue and intercellular junctions, such as the AJ, must be well maintained; however, they have been reported to be dissolved by several permeable factors. In particular, strongly permeable factors, such as HDIL-2, significantly increase actin stress fibers and induce endothelial cell permeability. However, our results demonstrated that the administration of CU06-1004 together with IL-2 injection reduced the formation of actin stress fibers and inhibited vascular leakage through F-actin and VE-cadherin staining. In summary, the abnormal permeability caused by IL-2-induced endothelial cell damage *in vitro* was restored by CU06-1004 treatment.

Although the anticancer effect of IL-2 immunotherapy has been reported to be excellent for a long time, continuous administration is impossible because of serious side effects such as VLS (Pires et al., 2021). Therefore, we constructed an IL-2-induced VLS mouse model to enable long-term administration of IL-2 immunotherapy by reducing the side effects of IL-2 in patients. To confirm the permeability of the IL-2-induced VLS model, we injected Evans blue. To confirm permeability in the IL-2-induced VLS model, Evans blue was injected into the tail vein. Our results showed that blue dye leakage from the tissue and skin induced by IL-2 was limited by the vascular stabilization induced by CU06-1004. Thus, combination therapy with CU06-1004 in a mouse model suggests the possibility of long-term administration of IL-2 immunotherapy by alleviation of VLS.

However, although the VLS-reducing effect of CU06-1004 in normal mice is interesting, it is essential to observe its impact in a tumor-bearing mouse model for potential clinical use. Therefore, we proceeded with the co-administration of IL-2 and CU06-1004 from day 7 after injecting the B16F10 melanoma tumor into the mice. Interestingly, the Evans blue staining results revealed a reduction in vascular leakage in tumor-bearing mice treated with the combination of IL-2 and CU06-1004. This result suggests the possibility of improving the side effects of IL-2 drugs in cancer patients.

Additionally, we aimed to analyze whether the CU06-1004 and IL-2 combination group could maintain or enhance the anti-cancer effect of IL-2 while inhibiting VLS. To do so, we investigated tumor growth and size between the groups in a tumor-bearing mouse model. Surprisingly, the combination treatment with CU06-1004 demonstrated the same extent of tumor size suppression as the treatment with IL-2 alone, and in some cases, it even enhanced the suppression. Consequently, we compared the number of immune cells expected to influence tumor growth and size changes. We found that changes in tumor size correlated with the number of cytotoxic CD8^+^ T cells in both the IL-2-alone and CU06-1004-combined treatment groups.

Next, we analyzed the expression of pro-inflammatory cytokines that are expected to be influenced by cytotoxic CD8^+^ T cells, using ELISA. As anticipated, the injection of IL-2 significantly increased the expression of IFNγ in mouse serum, indicating an immune activation response. Importantly, these results were also observed in the group administered the combination therapy with CU06-1004, suggesting that co-administration does not compromise the immunomodulatory effects of IL-2.

In conclusion, the co-administration of CU06-1004 and IL-2 shows promise for cancer treatment. It not only mitigates vascular leakage, which is a crucial concern with IL-2 treatment but also maintains or enhances the anti-cancer efficacy of IL-2. These findings highlight the potential of combination therapy with CU06-1004 to provide a more effective and long-term treatment option for cancer patients.

## 5 Conclusion

We demonstrate the suppression of high-dose IL-2-induced side effects and the improvement of anti-cancer effects through combined CU06-1004 and IL-2. In other words, a combination of CU06-1004 and IL-2 drugs is a new promising strategy to reduce severe VLS and maintained the immune response to cancer for a long time.

## Data Availability

The raw data supporting the conclusion of this article will be made available by the authors, without undue reservation.

## Abbreviations

1004, CU06-1004; IL-2, Interleukin 2; VLS, Vascular leaky syndrome; CLS, Capillary leaky syndrome; AJ, adherent junction.
